# Evaluation of the prognostic role of centromere 17 gain and HER2/topoisomerase II alpha gene status and protein expression in patients with breast cancer treated with anthracycline-containing adjuvant chemotherapy: pooled analysis of two Hellenic Cooperative Oncology Group (HeCOG) phase III trials

**DOI:** 10.1186/1471-2407-13-163

**Published:** 2013-03-28

**Authors:** George Fountzilas, Urania Dafni, Mattheos Bobos, Vassiliki Kotoula, Anna Batistatou, Ioannis Xanthakis, Christos Papadimitriou, Ioannis Kostopoulos, Triantafillia Koletsa, Eleftheria Tsolaki, Despina Televantou, Eleni Timotheadou, Angelos Koutras, George Klouvas, Epaminontas Samantas, Nikolaos Pisanidis, Charisios Karanikiotis, Ioanna Sfakianaki, Nicholas Pavlidis, Helen Gogas, Helena Linardou, Konstantine T Kalogeras, Dimitrios Pectasides, Meletios A Dimopoulos

**Affiliations:** 1 Department of Medical Oncology, “Papageorgiou” Hospital, Aristotle University of Thessaloniki School of Medicine, Thessaloniki, Greece; 2 Laboratory of Biostatistics, University of Athens School of Nursing, Athens, Greece; 3 Laboratory of Molecular Oncology, Hellenic Foundation for Cancer Research, Aristotle University of Thessaloniki School of Medicine, Thessaloniki, Greece; 4 Department of Pathology, Aristotle University of Thessaloniki School of Medicine, Thessaloniki, Greece; 5 Department of Pathology, Ioannina University Hospital, Ioannina, Greece; 6 Department of Clinical Therapeutics, “Alexandra” Hospital, University of Athens School of Medicine, Athens, Greece; 7 Division of Oncology, Department of Medicine, University Hospital, University of Patras Medical School, Patras, Greece; 8 Second Department of Medical Oncology, “Metropolitan” Hospital, Athens, Greece; 9 Third Department of Medical Oncology, “Agii Anargiri”, Cancer Hospital, Athens, Greece; 10 Department of Medical Oncology, IKA Hospital, Thessaloniki, Greece; 11 Department of Medical Oncology, 424 Army General Hospital, Thessaloniki, Greece; 12 Department of Medical Oncology, Ioannina University Hospital, Ioannina, Greece; 13 First Department of Medicine, “Laiko” General Hospital, University of Athens School of Medicine, Athens, Greece; 14 First Department of Medical Oncology, “Metropolitan” Hospital, Athens, Greece; 15 Translational Research Section, Hellenic Cooperative Oncology Group, Data Office, Athens, Greece; 16 Oncology Section, Second Department of Internal Medicine, “Hippokration” Hospital, Athens, Greece

**Keywords:** HER2, TOP2A, TopoIIa, Prognostic factors, Predictive factors, Adjuvant chemotherapy, Anthracyclines, Taxanes, Breast cancer

## Abstract

**Background:**

The *HER2* gene has been established as a valid biological marker for the treatment of breast cancer patients with trastuzumab and probably other agents, such as paclitaxel and anthracyclines. The *TOP2A* gene has been associated with response to anthracyclines. Limited information exists on the relationship of *HER2*/*TOP2A* gene status in the presence of centromere 17 (CEP17) gain with outcome of patients treated with anthracycline-containing adjuvant chemotherapy.

**Methods:**

Formalin-fixed paraffin-embedded tumor tissue samples from 1031 patients with high-risk operable breast cancer, enrolled in two consecutive phase III trials, were assessed in a central laboratory by fluorescence in situ hybridization for *HER2*/*TOP2A* gene amplification and CEP17 gain (CEP17 probe). Amplification of *HER2* and *TOP2A* were defined as a gene/CEP17 ratio of >2.2 and ≥2.0, respectively, or gene copy number higher than 6. Additionally, HER2, TopoIIa, ER/PgR and Ki67 protein expression was assessed by immunohistochemistry (IHC) and patients were classified according to their IHC phenotype. Treatment consisted of epirubicin-based adjuvant chemotherapy followed by hormonal therapy and radiation, as indicated.

**Results:**

*HER2* amplification was found in 23.7% of the patients and *TOP2A* amplification in 10.1%. In total, 41.8% of *HER2*-amplified tumors demonstrated *TOP2A* co-amplification. The median (range) of *HER2*, *TOP2A* and CEP17 gain was 2.55 (0.70-45.15), 2.20 (0.70-26.15) and 2.00 (0.70-26.55), respectively. Forty percent of the tumors had CEP17 gain (51% of those with *HER2* amplification). Adjusting for treatment groups in the Cox model, *HER2* amplification, *TOP2A* amplification, CEP17 gain and *HER2*/*TOP2A* co-amplification were not associated with time to relapse or time to death.

**Conclusion:**

*HER2* amplification, *TOP2A* amplification, CEP17 gain and *HER2*/*TOP2A* co-amplification were not associated with outcome in high-risk breast cancer patients treated with anthracycline-based adjuvant chemotherapy.

**Trial registration:**

Australian New Zealand Clinical Trials Registry (ANZCTR) ACTRN12611000506998 and ACTRN12609001036202

## Background

Breast cancer is the most frequent non-skin malignancy and the second leading cause of cancer death in American and European women [1,2]. Adjuvant chemotherapy is administered to most patients with high-risk operable breast cancer, since it prolongs disease-free survival (DFS) and overall survival (OS) [3]. Anthracyclines and taxanes are considered to be two of the most efficient drugs in this setting [4,5]. Despite intensive clinical research devoted to the role of adjuvant chemotherapy, the majority of patients do not benefit from its use and a small but considerable percentage of them suffer from long-term life-threatening toxicities, such as acute leukemia, myelodysplasia or irreversible congestive heart failure [6,7]. To select candidate patients for such aggressive treatments, robust prognostic markers in human breast cancer are needed. Investigators intensively evaluate well-established oncogenes or chromosome aberrations, using large tumor repositories, in an effort to widen their knowledge on the molecular mechanisms, gene interrelationships or gene function underlying breast cancer.

It has long been established that breast cancer is often characterized by gains or losses of specific chromosomes, leading to activation of oncogenes or inactivation of tumor suppressor genes [8]. Chromosome 17 is the second most gene-dense chromosome in the human genome, housing important genes for breast cancer pathophysiology, such as *BRCA1, HER2, RAD51C, RARA, TOP2A* and *TP53*[9]. Changes of chromosome 17 copy number (aneusomy) are extremely frequent in breast cancer [10]. These chromosome aberrations (reviewed in ref. [11]) are tightly linked to important cell functions, such as proliferation, apoptosis, angiogenesis and motility. Increased numbers of centromere 17 copies are seen in 10% to 50% of breast tumors [12-14], depending on the criteria used, and this is more common in tumors with *HER2* gene amplification. However, it has to be stated that an increase in chromosome 17 signals seen with fluorescence in situ hybridization (FISH) does not always correspond to true polysomy of the whole chromosome, but may rather represent a focal pericentromeric gain or partial polysomy [15]. Other abnormalities of chromosome 17 include losses and gains of genetic material in both the p and q arms, focal copy number gains and losses and other structural rearrangements [15,16]. Indeed, recent studies using different techniques, such as comparative genomic hybridization (CGH) [17,18], multiplex ligation-dependent probe amplification (MLPA) [19], single nucleotide polymorphism arrays (SNP arrays) [15], or FISH using alternative chromosome 17 reference genes (*RARA, TP53, SMS*) [20] suggested that true chromosome 17 polysomy is a rare event in breast cancer. In fact, in most of the cases, polysomy, as detected by FISH or chromogen in situ hybridization (CISH), actually reflects a gain or amplification in the pericentromeric region of the chromosome [21]. For these reasons the term “CEP17 gain” instead of “chromosome 17 polysomy”, is used here, referring to its detection by the centromere 17 enumeration probe (CEP17 probe).

CEP17 gain has been incriminated for the inconsistencies seen in cases with *HER2* gene amplification defined by absolute gene copy numbers, versus gene amplification defined by the ratio of *HER2* gene copy number to CEP17. Misclassification of *HER2* gene status based on dual-color FISH assays, due to CEP17 gain, may have important therapeutic implications since a number of patients considered being HER2-negative by the second definition could be denied trastuzumab.

Importantly, recently published data from retrospectively assessed (although prospectively collected) tumors, by triple color FISH, from 1762 patients who participated in the National Epirubicin Adjuvant Trial (NEAT/BR9601) suggested that CEP17 duplication was associated with increased relapse-free and overall survival in patients treated with an anthracycline compared to CMF [22].

The *HER2* oncogene is located on the long arm of chromosome 17 (17q12) [23]. *HER2* amplification and/or protein overexpression has been identified in 15% to 25% of invasive breast tumors [24] and is associated with worse prognosis [25]. *HER2* gene amplification has been shown to predict benefit from the use of several chemotherapeutic agents, including anthracyclines and paclitaxel [26,27]. Notably, a meta-analysis provided compelling evidence that the use of anthracyclines benefits exclusively those patients with *HER2* amplification [28]. However, other investigators could not confirm these data [29,30], suggesting that other genes, also located on chromosome 17, may regulate anthracycline responsiveness [26].

One such gene is the topoisomerase II alpha gene (*TOP2A*), which is located ~700 kb telomerically to *HER2* and encodes the alpha isozyme of the human topoisomerase II. In general, topoisomerases are responsible for transcription, replication and chromosome condensation and segregation during cell division [31,32]. *TOP2A* in particular is considered a molecular target for anthracyclines and other chemotherapeutic agents [33,34]. The *TOP2A* gene is amplified in 30%-40% of the tumors with *HER2* gene amplification, while deletions are frequently observed [35]. TOP2A gene amplification [36] and, perhaps, topoisomerase II alpha (TopoIIa) protein overexpression [37] may benefit high-risk breast cancer patients treated with anthracyclines.

Information regarding the interaction of *HER2/TOP2A* gene status in the presence of CEP17 gain with the outcome of breast cancer patients is limited. This urged us to investigate the prognostic role of HER2 and TopoIIa protein expression, as well as *HER2* and *TOP2A* gene status along with CEP17 gain in a large cohort of breast cancer patients. This is a prospective-retrospective study as described by Simon [38], performed in the context of two randomized, consecutively conducted, phase III trials (HE10/97 and HE10/00) with epirubicin-based adjuvant chemotherapy with or without paclitaxel [39-42].

## Methods

### Clinical studies

The HE10/97 trial [39] was a randomized phase III trial (ACTRN12611000506998) in patients with high-risk node-negative or intermediate/high-risk node-positive operable breast cancer, comparing four cycles of epirubicin (E) followed by four cycles of intensified CMF (E-CMF) with three cycles of E, followed by three cycles of paclitaxel (T, Taxol®, Bristol Myers-Squibb, Princeton, NJ) followed by three cycles of intensified CMF (E-T-CMF). All cycles were given every two weeks with G-CSF support. Dose intensity of all drugs in both treatment arms was identical, but cumulative doses and duration of chemotherapy period differed. Totally, 595 eligible patients entered the study in a period of 3.5 years (1997–2000).

The HE10/00 trial [40,41] was a randomized phase III trial (ACTRN12609001036202), in which patients were treated with E-T-CMF (exactly as in the HE10/97 trial) or with four cycles of epirubicin/paclitaxel (ET) combination (given on the same day) every three weeks followed by three cycles of intensified CMF every two weeks (ET-CMF). By study design, the cumulative doses and the chemotherapy duration were identical in the two arms but dose intensity of epirubicin and paclitaxel was double in the E-T-CMF arm. A total of 1086 eligible patients with node-positive operable breast cancer were accrued in a period of 5 years (2000–2005).

HER2-positive patients received trastuzumab upon relapse, as previously described [43]. Treatment schedules for the two studies, baseline characteristics and clinical outcomes of both trials have already been described in detail [39-42]. Primary tumor diameter, axillary nodal status and tumor grade were obtained from the pathology report. Clinical protocols were approved by local regulatory authorities, while the present translational research protocol was approved by the Bioethics Committee of the Aristotle University of Thessaloniki School of Medicine. All patients signed a study-specific written informed consent before randomization, which in addition to giving consent for the trial allowed the use of biological material for future research purposes.

### Tissue microarray (TMA) construction

**Figure 1:**
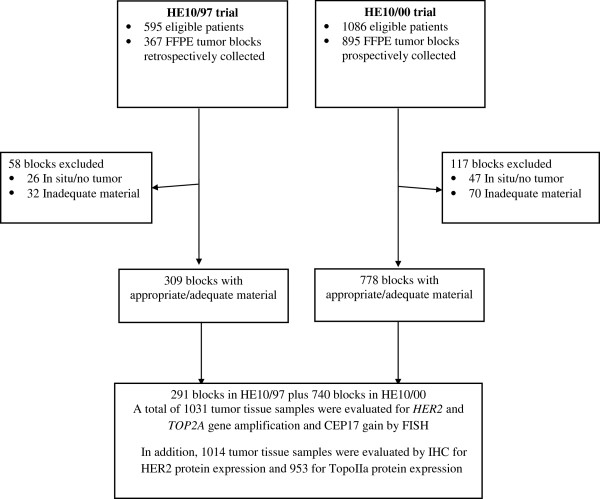
REMARK diagram.

Formalin-fixed paraffin-embedded (FFPE) tumor tissue samples from 1031 patients (61.3% of 1681 randomized patients) were collected from both trials, retrospectively in the first (HE10/97) and prospectively in the second (HE10/00). The REMARK diagram [44] for the study is shown in Figure 1. Hematoxylin-eosin stained sections from the tissue blocks were reviewed by two experienced breast cancer pathologists (M.B. and D.T.) and the most representative tumor areas were marked for the construction of the TMA blocks with the use of a manual arrayer (Model I, Beecher Instruments, San Prairie, WI), as previously described [45,46]. Each case was represented by 2 tissue cores, 1.5 mm in diameter, obtained from the most representative areas of primary invasive tumors or in some cases (9.6%) from synchronous axillary lymph node metastases and re-embedded in 51 microarray blocks. Each TMA block contained 38 to 66 tissue cores from the original tumor tissue blocks, while cores from various neoplastic, non-neoplastic and reactive tissues were also included, serving as controls for slide-based assays. Cases not represented, damaged or inadequate on the TMA sections were re-cut from the original blocks and these sections were used for protein and gene analysis. Histological grade was evaluated according to the Scarff, Bloom and Richardson system.

### Immunohistochemistry (IHC)

Immunohistochemical labeling was performed according to standard protocols on serial 2.5 μm thick sections from the original blocks or the TMA blocks. All cases were also stained for vimentin (clone V9, Dako, Glostrup, Denmark) and cytokeratin 8/18 (clone 5D3, Novocastra™, Leica Biosystems, Newcastle, U.K), which were used as control stains for tissue immunoreactivity and fixation, as well as identification of tumor cells. Tissue samples negative for the above antibodies were excluded from the study. To assure optimal reactivity, immunostaining was applied 7 to 10 days after sectioning at the Laboratory of Molecular Oncology of the Hellenic Foundation for Cancer Research, Aristotle University of Thessaloniki School of Medicine. The staining procedures for HER2 (A0485 polyclonal antibody, Dako), estrogen receptor (ER, clone 6 F11, Novocastra™, Leica Biosystems), progesterone receptor (PgR, clone 1A6, Novocastra™, Leica Biosystems) and Ki67 (clone MIB-1, Dako) were performed using a Bond Max™ autostainer (Leica Microsystems, Wetzlar, Germany), as previously described [47]. TopoIIa protein expression was evaluated using the KiS1 monoclonal antibody (Dako), as previously described [48] with slight modifications (antibody dilution: 1:200; detection system: Envision™, Dako).

### Interpretation of the IHC results

The evaluation of all IHC sections was done by two experienced breast cancer pathologists (M.B. and A.B.), blinded as to the patients’ clinical characteristics and survival data, according to existing established criteria, as previously described [43]. Briefly, HER2 protein expression was scored in a scale from 0 to 3+, the latter corresponding to uniform, intense membrane staining in >30% invasive tumor cells [49]; ER and PgR were evaluated using the Histoscore method (max score: 400) and were considered positive if staining was present in ≥1% of tumor cell nuclei [50]; for Ki67, the expression was defined as low (<14%) or high (≥14%) based on the percentage of stained/unstained nuclei from the tumor areas [51]; and, for TopoIIa immunostaining, a tumor was considered to be positive if moderate to intense nuclear staining was detected in >5% of tumor cells [52]. The mean percentage of stained cells from the two cores was calculated, while in cases with different intensities, the higher intensity score obtained from the two cores was used. If one of the tissue cores was lost or damaged the overall score was determined from the remaining one. When whole tissue sections were used, the entire tumor area was evaluated.

### Fluorescence in situ hybridization (FISH)

TMA sections or whole sections (5 μm thick) were cut for FISH analysis, using the ZytoLight® SPEC *HER2*/*TOP2A*/CEP17 triple-color probe kit (ZytoVision, Bremerhaven, Germany). The FISH was performed according to the manufacturer’s protocol with minor modifications. Four carcinoma cell lines (MDA-MB-231, MDA-MB-175, MDA-MB-453 and SK-BR-3) from the Oracle HER2 Control Slide (Leica Biosystems), with a known *HER2* gene status, were also used as a control for the FISH assays and analyzed for *HER2* and *TOP2A* genomic status.

For all probes, sequential (5 planes at 1.0 μm) digital images were captured using the Plan Apo VC 100x/1.40 oil objective (Nikon, Japan) using specific filters for each probe. The resulting images were reconstructed using specifically developed software for cytogenetics (XCyto-Gen, ALPHELYS, Plaisir, France). Processed sections were considered eligible for FISH evaluation according to the ASCO/CAP criteria [49]. For the evaluation of *HER2*/*TOP2A*/CEP17 status, non-overlapping nuclei from the invasive part of the tumor were randomly selected, according to morphological criteria using DAPI staining, and scored (M.B and E.T). The virtual slides of HER2, ER or PgR stains, created as previously described [47], were used for selecting the invasive part of the tumor in each TMA. Twenty tumor nuclei were counted according to Press et al. [53]. The *HER2* gene was considered to be amplified when the *HER2*/CEP17 ratio was >2.2 [49], or the mean *HER2* copy number was >6 [54] and deleted when the ratio was <0.8.

The *TOP2A* gene was considered to be amplified when the *TOP2A*/CEP17 ratio was ≥2.0 and deleted when the ratio was <0.8 [36]. Cases with ≥3 CEP17 hybridization signals detected in >30% of counted nuclei were classified as CEP17 gain [55]. Re-classification of CEP17 gain was performed for the current analysis in comparison to the previous report [56].

In cases with ratios at or near the cut-off (1.8-2.2 for amplifications and 0.7-0.9 for deletions), additional 20 or 40 nuclei were counted and the ratio was recalculated. In cases with a borderline ratio at 60 nuclei, additional FISH assays were performed in whole sections [57]. In addition, tumors were classified according to the number of gene copies as normal (≤4 copies), low gain (4–6 copies) or high gain (>6 copies) tumors. The first category included tumors with possible gene losses, diploid, or with replicated DNA; the second, tumors with possible polysomy for the gene of interest; and, the third, tumors with unequivocal gene amplification. All primary image data of the TMA and whole tumor sections have been digitally scanned and made publicly available at: http://www.hecog-images.gr/HER2/TOP2A/CEN17/FISH_HE10/97_HE10/00.

### Statistical analysis

Categorical data are presented as numbers and corresponding percentages, while continuous data are presented as median and range values. The Fisher’s exact or Pearson chi-square tests were used for group comparison of categorical data, while for continuous data the Mann–Whitney test was used. DFS was defined as the time interval from study entry to first locoregional recurrence, first distant metastasis, contralateral breast cancer, secondary neoplasm, death from the disease, or death from any cause, whichever occurred first [58]. OS was measured from study entry until death from any cause. Surviving patients were censored at the date of last contact. Kaplan-Meier curves and log-rank tests were used for comparing time to event distributions.

Cox proportional hazard regression analyses, adjusted for treatment, were performed for the examined markers, as well as for the combination of *HER2*/*TOP2A* gene status to assess prognostic significance on DFS and OS. In multivariate analysis, a backward selection procedure with p > 0.10 as a removal criterion based on the likelihood ratio test was performed to identify significant clinicopathological variables among the following: age (≥50 vs. <50), treatment group (E-CMF vs. ET-CMF vs. E-T-CMF), menopausal status (postmenopausal vs. premenopausal), histological grade (III-undifferentiated vs. I-II), Ki67 protein expression (high vs. low), tumor size (>5 cm vs. 2 to 5 cm vs. <2 cm), number of positive axillary nodes (≥4 vs. 0–3), ER/PgR status (positive vs. negative), adjuvant hormonotherapy (yes vs. missing vs. no) and type of operation (breast-conserving surgery vs. modified radical mastectomy). Treatment group and the examined markers were included in the final model, in order to examine whether they added independent prognostic information to the model containing the significant clinicopathological parameters.

The results of this study are presented according to reporting recommendations for tumor marker prognostic studies [44]. No adjustments for multiple comparisons were done. Statistical analyses were performed using the following statistical software: SPSS for Windows (version 15.0, IBM Corporation, Armonk, NY) and SAS for Windows (version 9.3, SAS Institute Inc., Cary, NC).

## Results

A total of 1031 patients with available FFPE tumor tissue blocks were included in the analysis at a median follow-up of 106 months (updated in March 2012). Results at median follow-up of 92 months were presented at the 2011 San Antonio Breast Cancer Symposium [56].

Selected patient and tumor characteristics are presented in Table 1. The majority of the patients were postmenopausal (53.1%), had ≥4 positive nodes (60.4%) and tumors of ductal histology (77.6%), while approximately half of the patients had tumors of high grade (50.2%). The basic clinicopathological characteristics were similar between patients with and without available tissue blocks in each study, except for the number of positive nodes, radiotherapy treatment and histological grade (above II) (Additional file 1: Table S1). Patients with available tissue blocks had a higher incidence of ≥4 positive nodes (p = 0.022 and p = 0.027 for HE10/97 and HE10/00, respectively). This fact was probably reflected in the corresponding more frequent use of radiotherapy treatment (p = 0.024 and p = 0.021), while higher histological grade was more frequent in patients with available blocks only in the HE10/00 trial (p = 0.026).

**Table 1:** Selected patient and tumor characteristics

	**N = 1031**
Age in years	
Median (range)	52.5 (22–79)
Number of positive nodes	
Median (range)	4 (0–43)
	**N (%)**
Randomization group	
E-T-CMF	504 (48.9)
E-CMF	157 (15.2)
ET-CMF	370 (35.9)
Age	
<50	426 (41.3)
≥50	605 (58.7)
Menopausal status	
Premenopausal	484 (46.9)
Postmenopausal	547 (53.1)
Type of surgery	
MRM	706 (68.5)
Breast-conserving	325 (31.5)
Tumor size (cm)	
≤2	320 (31.0)
2.1-5	584 (56.6)
>5	127 (12.3)
Number of positive nodes	
0-3	408 (39.6)
≥4	623 (60.4)
Histological grade	
I-II	513 (49.8)
III-Undifferentiated	518 (50.2)
Histology type	
Ductal	800 (77.6)
Lobular	105 (10.2)
Mixed	73 (7.1)
Other	53 (5.1)
Radiotherapy	782 (75.8)
Hormonal therapy	799 (77.5)

MRM, modified radical mastectomy; N, number.

### Incidence and associations between examined biological markers

**Figure 2:**
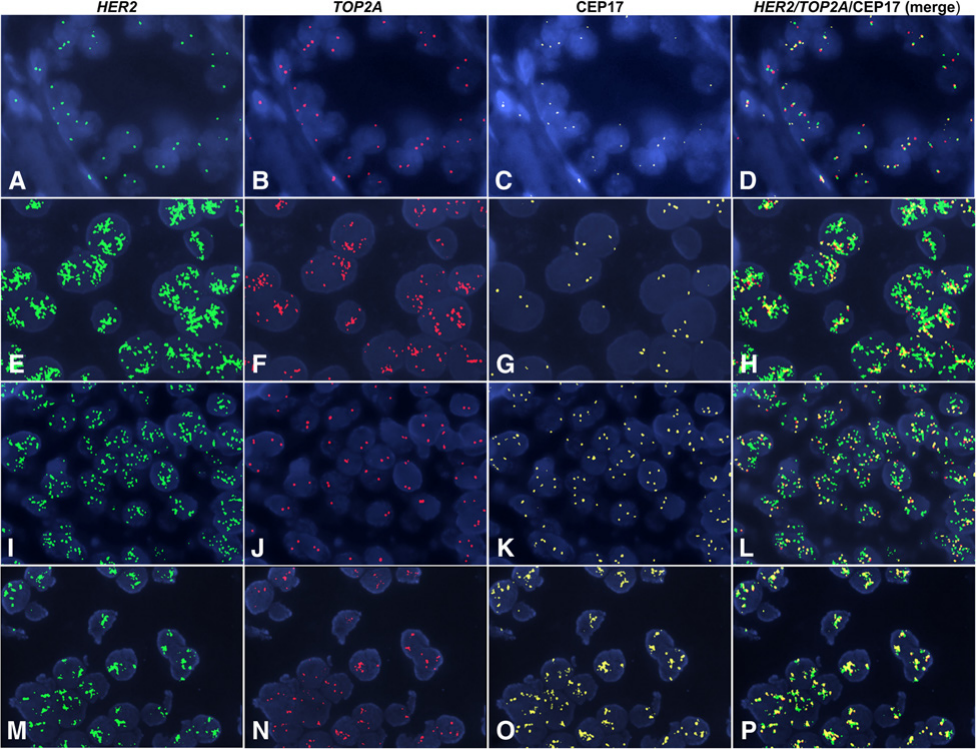
**Representative FISH images in invasive breast carcinoma (IBC) cases, using the *HER2*/ *TOP2A */CEP17 triple-color probes.** In the first four panels (**A-D**) an IBC case is shown with normal status of the *HER2* gene (**A**), *TOP2A* gene (**B**) and CEP17 (**C**). An IBC case (**E-H**) showing simultaneous amplification of the *HER2* and *TOP2A* genes (**E-F**), as well as CEP17 gain (**G**). The third IBC case presented in panels (**I-L**) showed amplification of the *HER2* gene (**I**), normal status of the *TOP2A* gene (**J**) and CEP17 gain (**K**). In the last case, co-amplification of the *HER2* (**M**) and *TOP2A* genes (**N**) was found in tumor cells, accompanied by high-level CEP17 gain (**O**). The last panel for each case (panels **D**, **H**, **L** and **P**) depicts a merged image of the three-colored probes. Magnification x1000. CEP17, centromere 17 enumeration probe.

**Figure 3:**
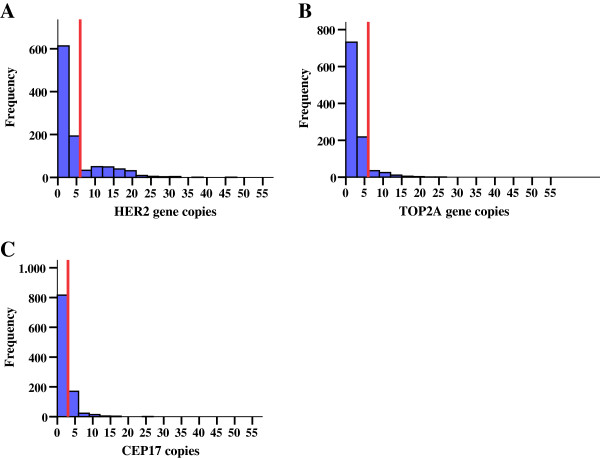
**Distribution of *HER2*, *TOP2A *and CEP17 copies (A, B and C).** Red line represents 6 gene copies (for **A** and **B**) and 3 copies for **C**.

Representative FISH images for *HER2*, *TOP2A* and CEP17 are shown in Figure 2. The distribution of centrally assessed tumor markers by FISH and IHC are presented in Table 2. Cases with *HER2* deletions (n = 27, 2.6%) were grouped together with *HER2* normal tumors, for analysis purposes. Amplification, classified according to gene/CEP17 ratios, was found for *HER2* in 23.7% and for *TOP2A* in 10.1% of the tumors. The incidence of amplified tumors was lower when amplification was considered according to the cut-off of >6 copies for each gene (Table 2). Ten cases were equivocal for *HER2* (with *HER2*/CEP17 ratios between 1.8-2.2 and ≤6 gene copies) and they were also included in the *HER2* normal tumors, for analysis purposes. CEP17 gain was seen in approximately 40% of tumors (Table 2). Histograms of the distribution of *HER2*, *TOP2A* and CEP17 copy numbers are presented in Figure 3.

**Table 2:** Distribution of centrally assessed tumor markers by FISH and IHC

		**N (%)**
***FISH***	CEP17 status (n = 1031)	
	Median (range)	2.00 (0.70-26.55)
	No gain	620 (60.1)
	Gain	411 (39.9)
	*HER2* (gene copies) (n = 1031)	
	Median (range)	2.55 (0.70-45.15)
	Low normal-replicated (≤4)	742 (72.0)
	Low gain (4–6)	65 (6.3)
	High gain (>6)	224 (21.7)
	*HER2* gene status (n = 1031)	
	Non-amplified^1^	787 (76.3)
	Amplified^2^	244 (23.7)
	*TOP2A* (gene copies) (n = 1031)	
	Median (range)	2.15 (0.70-26.15)
	Low normal-replicated (≤4)	875 (84.9)
	Low gain (4–6)	77 (7.5)
	High gain (>6)	79 (7.7)
	*TOP2A* gene status (n = 1031)	
	Deleted	52 (5.0)
	Non-amplified	875 (84.9)
	Amplified^3^	104 (10.1)
***IHC***	HER2 (n = 1014)	
	0	319 (31.5)
	1+	379 (37.4)
	2+	171 (16.9)
	3+	145 (14.3)
	TopoIIa (n = 953)	
	Negative	441 (46.3)
	Positive	512 (53.7)
	ER (n = 1018)	
	Negative	272 (26.7)
	Positive	746 (73.3)
	PgR (n = 1024)	
	Negative	335 (32.7)
	Positive	689 (67.3)
	Ki67 (n = 1000)	
	Low	322 (32.2)
	High	678 (67.8)

^1^27 cases (2.6%) had a deletion with a *HER2*/CEP17 ratio <0.8.

^2^204 cases (83.6%) with *HER2*/CEP17 ratio >2.2 and 40 cases (16.4%) with *HER2* gene gain (>6 copies).

^3^64 cases (61.5%) with *TOP2A*/CEP17 ratio ≥2.0 and 40 cases (38.5%) with *TOP2A* gene gain (>6 copies).

Examining the association of markers with clinicopathological parameters, CEP17 gene gain was found to be associated only with postmenopausal status (48.1% in no gain vs. 60.6% in gain, p < 0.001) (Additional file 1: Table S2). *HER2* gene amplification was associated with higher histological grade (45% in non-amplified vs. 66% in amplified, p < 0.001), ductal carcinoma (75% in non-amplified vs. 87% in amplified, p < 0.001), negative receptor status (16% in non-amplified vs. 44% in amplified, p < 0.001) and high Ki67 (64% in non-amplified vs. 79% in amplified, p < 0.001), while *TOP2A* amplification was associated with higher histological grade (58% in deleted vs. 49% in non-amplified vs. 62% in amplified, p = 0.023) and negative receptor status (37% in deleted vs. 20% in non-amplified vs. 40% in amplified, p < 0.001).

Overall, 24% of the patients had a *HER2*-positive status, based on either *HER2* gene/CEP17 ratio of >2.2 or gene copy number of >6 or an IHC score of 3+. Interestingly, 27 tumors with HER2 IHC scores of 0 (7 cases) or 1+ (20 cases), were found to be amplified either by gene gain >6 (n = 3) or FISH ratio >2.2 (n = 24) (Additional file 1: Table S3). In addition, among cases with HER2 IHC scores of 0 or 1+, there were 17 tumors (2.4%) with *HER2* deletion. It is worth noting that among 204 cases with *HER2*/CEP17 ratio >2.2 (i.e., amplified by ratio criteria), 184 (90%) also had >6 *HER2* gene copies (i.e., amplified by gene copy criteria).

Tumors with CEP17 gain were also *HER2* amplified in about one third of the cases (N = 120), while they were *TOP2A* amplified in 15% of the cases (N = 59) (Table 3). Among 244 *HER2* amplified tumors, 51% had CEP17 gain. Similar percentages were observed for CEP17 gain in *TOP2A* amplified (58%) and deleted tumors (65%). Overall, tumors with low *HER2* or *TOP2A* copy numbers had CEP17 gain in 37% and 36%, respectively (Table 3). In addition, among 827 tumors with *HER2*/CEP17 ratio ≤2.2, 327 (40%) had CEP17 gain. Among 10 equivocal cases with *HER2*/CEP17 ratios between 1.8-2.2 there was only one case with CEP17 gain.

**Table 3:** **CEP17 status according to *HER2 *and *TOP2A *gene copy number and amplification status**

	**CEP17 status**		
	**No gain**	**Gain**	
	**N (%)**	**N (%)**	**p**
***HER2 *gene copies**			<0.001
≤4	506 (68.2)	236 (31.8)	
4-6	10 (15.4)	55 (84.6)	
>6	104 (46.4)	120 (53.6)	
***TOP2A *gene copies**			<0.001
≤4	587 (67.1)	288 (32.9)	
4-6	13 (16.9)	64 (83.1)	
>6	20 (25.3)	59 (74.7)	
***HER2 *gene status**			<0.001
Non-amplified	500 (63.5)	287 (36.5)	
Amplified	120 (49.2)	124 (50.8)	
***TOP2A *gene status**			<0.001
Deleted	18 (34.6)	34 (65.4)	
Non-amplified	558 (63.8)	317 (36.2)	
Amplified	44 (42.3)	60 (57.7)	

The distribution of *TOP2A* and CEP17 by breast cancer tumor subtypes is presented in Table 4. Among 126 triple-negative breast cancer (TNBC) tumors, no amplifications of *TOP2A* were found. CEP17 gain was more frequent in Luminal-HER2 and HER2-enriched tumors.

**Table 4:** ***HER2*, *TOP2A *and CEP17 status and TopoIIa protein expression according to breast cancer subtypes defined by immunohistochemistry**

	**Luminal A**	**Luminal B**	**Luminal-HER2**	**HER2-enriched**	**TNBC**
	**N (%)**	**N (%)**	**N (%)**	**N (%)**	**N (%)**
**FISH**					
***HER2 *gene status**					
Non-amplified	242 (100.0)	386 (100.0)	3 (2.2)	1 (0.9)	126 (100.0)
Amplified	0	0	135 (97.8)	107 (99.1)	0
***TOP2A *gene status**					
Deleted	5 (2.1)	15 (3.9)	12 (8.7)	11 (10.2)	8 (6.3)
Non-amplified	236 (97.5)	371 (96.1)	65 (47.1)	55 (50.9)	118 (93.7)
Amplified	1 (0.4)	0	61 (44.2)	42 (38.9)	0
**CEP17 status**					
No gain	149 (61.6)	234 (60.6)	70 (50.7)	50 (46.3)	93 (73.8)
Gain	93 (38.4)	152 (39.4)	68 (49.3)	58 (53.7)	33 (26.2)
**IHC**					
**TopoIIa**					
Negative	165 (73.7)	130 (35.2)	48 (36.9)	43 (41.7)	49 (42.6)
Positive	59 (26.3)	239 (64.8)	82 (63.1)	60 (58.3)	66 (57.4)

TNBC, triple-negative breast cancer.

Patients were classified as: luminal A (ER-positive and/or PgR-positive, HER2-negative, Ki67^low^); luminal B (ER-positive and/or PgR-positive, HER2-negative, Ki67^high^); luminal-HER2 (ER-positive and/or PgR-positive, HER2-positive); HER2-enriched (ER-negative, PgR-negative, HER2-positive); and TNBC (ER-negative, PgR-negative, HER2-negative).

Associations of *TOP2A* gene status and TopoIIa protein expression are shown in detail (Additional file 1: Table S4). *TOP2A* deletions did not result in lower TopoIIa expression. Among 953 cases with paired *TOP2A* gene status and protein expression data, there were 28 tumors with *TOP2A* gene deletion and simultaneous protein expression. No association was found between TopoIIa protein expression and *TOP2A* gene amplification (p = 0.22).

Significant associations were observed between CEP17 gene status and HER2 protein expression, as well as TopoIIa protein expression (Additional file 1: Table S5). More specifically, CEP17 gain was more frequent in HER2 2+ and 3+ tumors and in tumors expressing TopoIIa.

In total, 42% of *HER2* amplified tumors demonstrated *TOP2A* co-amplification (Additional file 1: Table S6). Among the *HER2* non-amplified cases, 28 deletions (3.6%) and only two amplifications of the *TOP2A* gene were identified.

### Associations of examined markers with prognosis

DFS and OS did not differ significantly between treatment groups. At a median follow-up of 106 months (range 0.1-167), the 5-year DFS rates were 75%, 69% and 75%, while the OS rates were 88%, 81% and 86%, for the E-T-CMF, E-CMF and ET-CMF groups, respectively (Additional file 1: Table S7).

**Figure 4:**
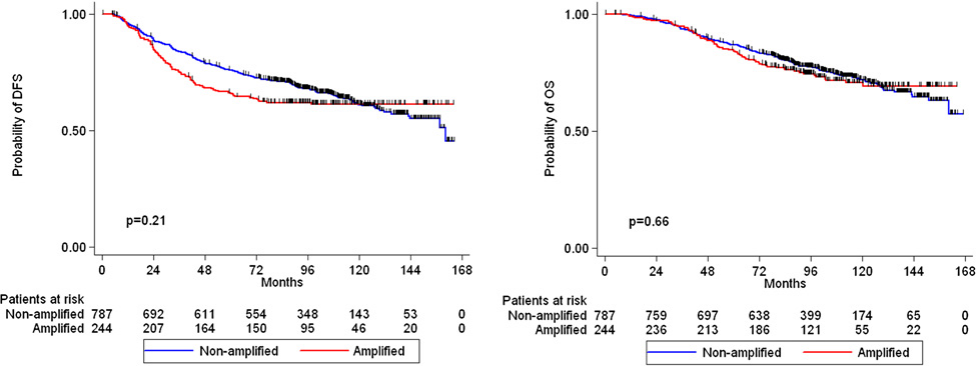
**Kaplan-Meier curves for DFS and OS according to *HER2 *gene status (log-rank test p-values).**

**Figure 5:**
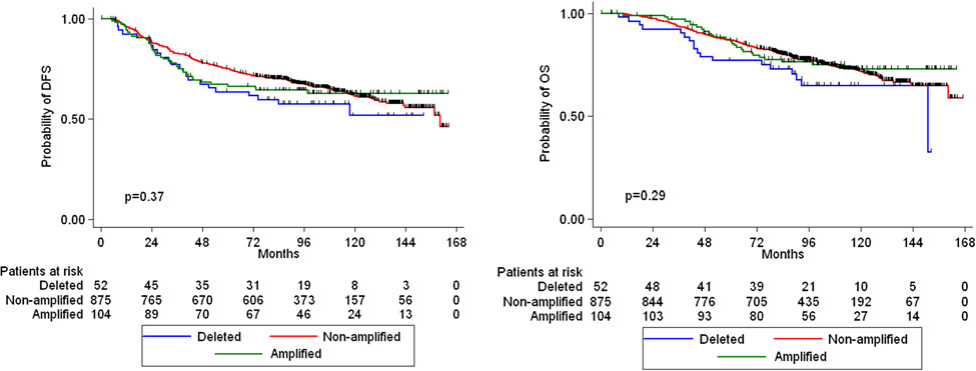
**Kaplan-Meier curves for DFS and OS according to *TOP2A *gene status (log-rank test p-values).**

**Figure 6:**
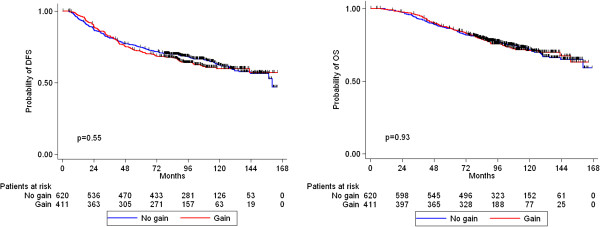
Kaplan-Meier curves for DFS and OS according to CEP17 gain (log-rank test p-values).

**Figure 7:**
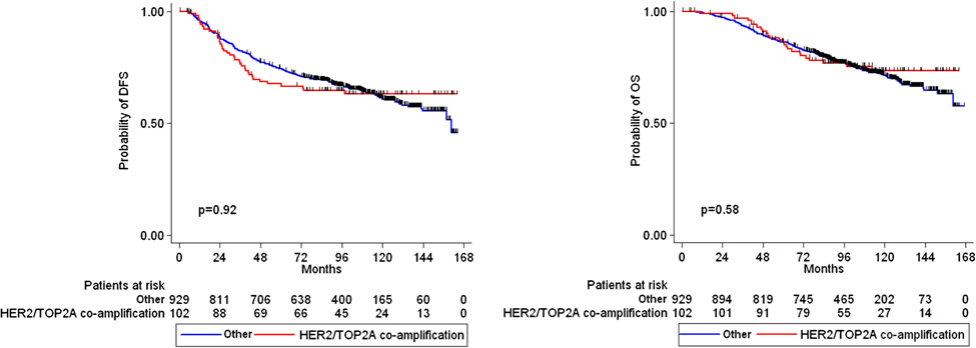
**Kaplan-Meier curves for DFS and OS according to *HER2/TOP2A *co-amplification (log-rank test p-values).**

*HER2* amplification, *TOP2A* amplification, TopoIIa protein expression, CEP17 gain and *HER2*/*TOP2A* co-amplification were not associated with either relapse or death (Figures 4, 5, 6 and 7). Similarly, when examining combined *TOP2A* gene pathology (deletion and amplification) vs. normal *TOP2A*, no effect on patient outcome was observed. This did not change when adjusting for treatment group in the Cox regression model. *HER2* and *TOP2A* gene copy numbers (amplified vs. low gain vs. low-normal-replicated) were also not associated with DFS or OS. Stratifying by CEP17 status, differences in outcome by *HER2* gene status (amplified vs. non-amplified tumors) and by *TOP2A* gene status (amplified vs. deleted vs. non-amplified tumors) were examined. No such differences were observed for either DFS or OS. The predictive role of all examined markers for paclitaxel treatment were also evaluated, performing Cox model analysis with interaction terms of each gene with treatment (paclitaxel vs. no paclitaxel). None of the markers tested was predictive for paclitaxel treatment.

**Figure 8:**
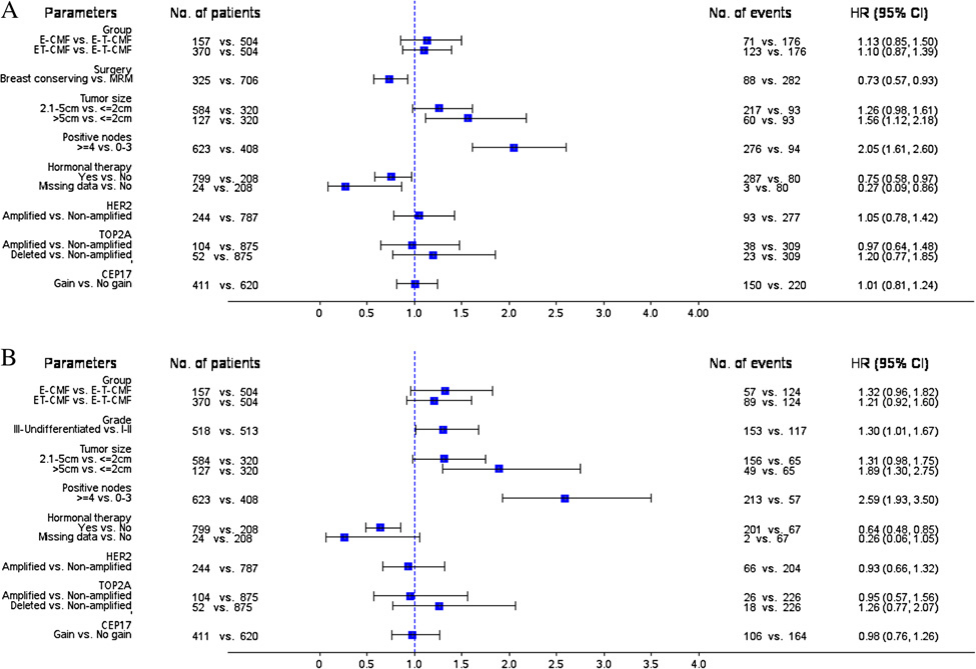
Multivariate analyses presented by forest plots in terms of DFS (A) and OS (B).

Multivariate analyses for the examined biological markers, in the presence of significant clinical parameters and treatment group, are presented by forest plots (Figure 8). Clinicopathological factors associated with increased risk for both relapse and death were tumor size of more than 5 cm (p = 0.009 for DFS and p = 0.001 for OS) and four or more positive nodes (p < 0.001 for both DFS and OS). Hormonal therapy was associated with improved DFS and OS (p = 0.028 and p = 0.002, respectively), while breast-conserving surgery was associated with improved DFS only (p = 0.011) and high histological grade with poor OS only (p = 0.039). No association was found with DFS or OS for any of the examined chromosome 17 markers. Finally, none of the examined markers were associated with either DFS or OS in the context of univariate or multivariate analyses, when excluding the lymph node samples.

## Discussion

In the present study we investigated the prognostic role of CEP17 gain in relation to *HER2* and *TOP2A* gene status and protein expression in 1031 patients with operable breast cancer. All these patients were treated with epirubicin-based adjuvant chemotherapy in the context of two consecutively conducted phase III trials [39-41]. In a previous study published by our group for the HE10/00 and HE10/97 cohorts [42], patients with either luminal B, luminal-HER2 or HER2-enriched tumors performed worse than those with luminal A tumors, while patients with triple-negative tumors had the worst outcome. In addition, it was observed that the HER2-enriched subtype was predictive of response to paclitaxel-containing treatments. These prognostic and predictive HER2-related effects were breast cancer subtype specific and were not maintained in the present study. An earlier observation reported for this cohort, of HER2 amplification being predictive for OS benefit from adjuvant treatment with paclitaxel [56], was not confirmed in the current analysis with updated follow-up. In both analyses however, the ability to detect any predictive impact of *HER2*/*TOP2A* amplification or CEP17 gain in the presence of taxanes was limited (only 1 of the 4 trial arms did not include taxanes). The present results concerning HER2 are in line with reports on the prognostic value of this marker [59,60]. A recent meta-analysis suggests that patients with both *HER2* amplified and non-amplified tumors may benefit from anthracyclines [61,62]. This could not be investigated in the current study, since all patients had been treated with anthracyclines.

Among *HER2* amplified tumors, 42% exhibited *TOP2A* co-amplification, which is within the reported range of 35%-50% for this genomic alteration [26,29,63,64]. *TOP2A* deletions were more common in *HER2* amplified tumors, comprising approximately 10% of the *HER2* amplified cases. *TOP2A* gene pathology (amplification, deletion and combinations of both) has been reported as a favorable prognostic and predictive marker in adjuvant-treated breast cancer patients [36,65]. However, in the present study we did not observe any association between patient outcome and *TOP2A* amplification, deletion, or both, in accordance with the recent meta-analysis mentioned above [62].

The clinical importance of CEP17 gain, as detected by FISH, in human breast cancer remains a controversial issue. From the biological perspective, CEP17 gain and chromosome 17 polysomy do not represent the same situation, since the first one corresponds to the fluorescent signals of a 5.6 kb region, while the second reflects aberrant numbers of the whole chromosome, which should be demonstrated with spectral karyotyping (SKY) or other cytogenetic approaches. The CEP17 FISH probe detects the alpha-satellite repeat region at the centromere of chromosome 17, at 17p11.1-17q11.1. The specificity of CEP17 remains undetermined, while this probe and a centromeric probe detecting additional neighboring regions on 17p11.2-12 yield different results concerning chromosome 17 status and, therefore, different *HER2* gene amplification status, when the latter is assessed as *HER2*/CEP17 ratio of >2.2 [66]. This may reflect the presence of the probed satellite repeats outside the centromeric region, which happen during evolution [67] and probably during cancer clone evolution, as well. Another problem for assessing low copy gains is that during DNA synthesis and in the G2/M phases, the targeted regions will appear double (three to four copies instead of two, taking into account the nuclear truncation effect during paraffin block sectioning). The cut-off used in the present study for the classification of CEP17 gains has been shown to correct for the maximally four centromeric signals that would be expected in this situation [55]. With this cut-off, we detected CEP17 gain in approximately 40% of all carcinomas examined, which is in the range of published results when using the FISH method in all-type breast carcinoma series (10-50%) [12-14,54,68,69].

Measurement of CEP17 probe signals reflects the condition of the corresponding centromeric area and can by no means reflect gains of the entire chromosome 17 or “chromosome 17 polysomy”, as often reported in the literature. In the same treatment settings, depending on how CEP17 signals are classified and interpreted, and also depending on the drugs administered, the effect of CEP17 status on patient outcome may vary. Thus, in the adjuvant setting, by using the same FISH probe, duplication of the CEP17 region of chromosome 17 seems to be predictive of benefit from anthracyclines [22,70] or of borderline association with clinical response to the same drugs [71]. Furthermore, CEP17 gain in the absence of *HER2*/*TOP2A* amplification has been reported by one recent study to be an unfavorable prognostic marker [72]; however, no prognostic value was identified for CEP17 gain in other studies [55,64,69], which is in line with our present findings.

CEP17 appears to be related with disease prognosis when this marker is combined with *HER2* status. Whether “polysomy” 17 drives *HER2* amplification or the opposite is true, as was recently suggested [54,73], remains unanswered; it is, however, noteworthy that “polysomy” 17 is rarely observed in circulating tumor cells from patients with metastatic breast cancer and when present, it corresponds to HER2-negative primary tumors [74]. In the absence of *HER2* amplification, CEP17 “polysomy” has been reported to confer a more favorable prognosis [75] or to be associated with aggravating prognostic markers [76]. In addition to these contradictory results, herein we did not observe any interaction between CEP17 and *HER2* status. Methodological differences in the assessment of these parameters and cohort-fitted results should account for the diversity of data regarding the role of CEP17/*HER2* status on adjuvant-treated breast cancer patients.

Although more than half of the tumors with CEP17 gain were not *HER2* or *TOP2A* amplified, we did observe a higher than double incidence of CEP17 gain in tumors with aberrant *HER2* and *TOP2A* genes (amplification, deletion, and, especially, high copy gains) in comparison to tumors with a normal status of these genes. These data are in line with “polysomy” 17 correlating with multiple copies of *HER2* but not with *HER2* amplification [77], while they further justify the higher incidence of CEP17 gain in the luminal-HER2 and HER2-enriched subtypes, as described in this study.

Equivocal HER2 IHC findings were observed in cases with chromosome 17 “polysomy” and correspondingly increased *HER2* gene copy numbers [54,78]. In the present study, the incidence of CEP17 gains was strongly related to HER2 IHC grades, but it did not contribute to the further assessment of HER2 IHC 2+ cases. With respect to FISH equivocal cases, herein we used very stringent criteria involving both gene/CEP17 ratios and gene copy numbers. Increased CEP17 ratios might result in false negative *HER2*/CEP17 ratios of ≤2.2; on the other hand, their coincidence with increased *HER2* copies is not equivalent to HER2-positive disease [79]. The few (n = 10, <0.01% of the entire tumor series) FISH equivocal tumors for *HER2* gene status were HER2 IHC 0 to 2+. CEP17 gain was observed in only one such case. Hence, at least in the present series, CEP17 gain did not aid further in the classification of equivocal *HER2* gene status cases. In addition, we observed that most of the tumors with low-gain of *HER2* and/or *TOP2A* copies indeed had CEP17 gains as well. However, in order to evaluate the impact of these concomitant alterations on patient outcome, larger patient series with this sub-category of tumors would be needed.

## Conclusions

The present combined chromosome 17 marker analysis by FISH represents one of the largest of its type in early high-risk breast cancer. With the cut-offs used for the characterization of CEP17 gain, as well as *HER2* and *TOP2A* gene amplification, these chromosome 17 markers, individually or in conjunction, did not appear to be related with patient outcome.

## Abbreviations

CGH: Comparative genomic hybridization; ANZCTR: Australian New Zealand Clinical Trials Registry; BRCA1: Breast cancer type 1 susceptibility protein; CEP17: Centromere 17 enumeration probe; CISH: Chromogen in situ hybridization; CMF: Cyclophosphamide, Methotrexate, Fluorouracil; DFS: Disease-free survival; DNA: Deoxyribonucleic acid; E: Epirubicin; ER: Estrogen receptor; FFPE: Formalin-fixed, paraffin-embedded; FISH: Fluorescence in situ hybridization; G-CSF: Granulocyte-colony stimulating factor; HeCOG: Hellenic Cooperative Oncology Group; HER2: Human epidermal growth factor receptor 2; HR: Hazard ratio; HT: Hormonal therapy; IHC: Immunohistochemistry; Ki67: Antigen Ki67; MLPA: Multiplex ligation-dependent probe amplification; OS: Overall survival; PgR: Progesterone receptor; RAD51C: RAD51 homolog C; RARA: Retinoic acid receptor, alpha; REMARK: Reporting Recommendations for Tumor Marker Prognostic Studies; RNA: Ribonucleic acid; SMS: Spermine synthase; SNP: Single nucleotide polymorphism; T: Taxol (Paclitaxel); TMA: Tissue microarray; TNBC: Triple-negative breast cancer; TOP2A: Topoisomerase II alpha (gene expression); TopoIIa: Topoisomerase II alpha (protein expression); TP53: Tumor protein 53.

## Competing interests

On behalf of the Hellenic Foundation for Cancer Research, Athens, Greece, the senior author (GF) has pending patent applications with Siemens Healthcare Diagnostics, Tarrytown, NY. The rest of the authors declare that they have no competing interests.

## Authors’ contributions

GF conceived of the study, participated in its design and coordination, contributed to the analysis and interpretation of data and drafted the manuscript. UD conceived of the study, participated in its design, contributed to the analysis and interpretation of data and drafted the manuscript. MB carried out the IHC and FISH analysis, contributed to the analysis and interpretation of data and drafted the manuscript. VK conceived of the study, participated in its design, carried out the molecular studies, contributed to the analysis and interpretation of data and drafted the manuscript. AB carried out the immunoassays and contributed to the analysis and interpretation of data. IX participated in the acquisition of data and contributed to the collection of the tumor tissue samples analyzed in the study. CP participated in the acquisition of data and contributed to the collection of the tumor tissue samples analyzed in the study. TK carried out the molecular studies and contributed to the analysis and interpretation of data. ET carried out the FISH assays and contributed to the analysis and interpretation of data. DT carried out the immunoassays and contributed to the analysis and interpretation of data. ET participated in the acquisition of data and contributed to the collection of the tumor tissue samples analyzed in the study. AK participated in the acquisition of data and contributed to the collection of the tumor tissue samples analyzed in the study. GK participated in the acquisition of data. ES participated in the acquisition of data and contributed to the collection of the tumor tissue samples analyzed in the study. NP participated in the acquisition of data and contributed to the collection of the tumor tissue samples analyzed in the study. CK participated in the acquisition of data and contributed to the collection of the tumor tissue samples analyzed in the study. IS participated in the acquisition of data. NP participated in the acquisition of data and contributed to the collection of the tumor tissue samples analyzed in the study. HG participated in the acquisition of data and contributed to the collection of the tumor tissue samples analyzed in the study. HL participated in the acquisition of data and contributed to the collection of the tumor tissue samples analyzed in the study. KTK conceived of the study, participated in its design, contributed to the analysis and interpretation of data and drafted the manuscript. DP conceived of the study, participated in its design and contributed to the analysis and interpretation of data. MAD conceived of the study, participated in its design, contributed to the analysis and interpretation of data and drafted the manuscript. All authors read and approved the final manuscript.

## Pre-publication history

The pre-publication history for this paper can be accessed here:

http://www.biomedcentral.com/1471-2407/13/163/prepub

## Supplementary Material

Additional file 1: Table S1 Comparison of basic clinicopathological characteristics between patients with and without available tissue material (paraffin blocks) per study. **Table S2.** Association of CEP17, *HER2* and *TOP2A* markers with basic clinicopathological parameters. **Table S3.** Distribution of *HER2* gene status according to HER2 protein expression. **Table S4.** Distribution of *TOP2A* gene status according to TopoIIa protein expression. **Table S5.** Distribution of CEP17 status according to HER2 and TopoIIa protein expression. **Table S6.** Association of *HER2* and *TOP2A* gene status assessed by FISH. **Table S7.** Survival data for the total study population and according to randomization group. Click here for file
