# Supplementary material for: Evaluation of the prognostic role of centromere 17 gain and HER2/topoisomerase II alpha gene status and protein expression in patients with breast cancer treated with anthracycline-containing adjuvant chemotherapy: pooled analysis of two Hellenic Cooperative Oncology Group (HeCOG) phase III trials
Source: BMC Cancer. 2013 Mar 28;13:163. doi: 10.1186/1471-2407-13-163 (PMC3621498; doi:10.1186/1471-2407-13-163)
Supplement: Additional file 1: Table S1 — Comparison of basic clinicopathological characteristics between patients with and without available tissue material (paraffin blocks) per study. Table S2. Association of CEP17, HER2 and TOP2A markers with basic clinicopathological parameters. Table S3. Distribution of HER2 gene status according to HER2 protein expression. Table S4. Distribution of TOP2A gene status according to TopoIIa protein expression. Table S5. Distribution of CEP17 status according to HER2 and TopoIIa protein expression. Table S6. Association of HER2 and TOP2A gene status assessed by FISH. Table S7. Survival data for the total study population and according to randomization group. [file 1471-2407-13-163-S1.doc]

**Table S1. Comparison of basic clinicopathological characteristics between patients with and without available tissue material (paraffin blocks) per study.**

|  | **HE10/97** | |  | **HE10/00** | |  |
| --- | --- | --- | --- | --- | --- | --- |
|  | **With block** | **Without block** |  | **With block** | **Without block** |  |
|  | **N=291** | **N=304** | **p** | **N=740** | **N=346** | **p** |
| Age in years  Median (range) | 51 (22-78) | 50 (24-75) | 0.31 | 53 (22-79) | 52 (24-77) | 0.26 |
| Number of positive nodes  Median (range) | 6 (0-43) | 5 (0-54) | 0.054 | 4 (0-40) | 3 (0-37) | 0.058 |
|  | **N (%)** | **N (%)** |  | **N (%)** | **N (%)** |  |
| Randomization group  E-T-CMF  E-CMF  ET-CMF | 134 (46.0)  157 (54.0)  - | 164 (53.9)  140 (46.1)  - | 0.059 | 370 (50.0)  -  370 (50.0) | 181 (52.3)  -  165 (47.7) | 0.52 |
| Age  <50  >50  Missing data | 139 (47.8)  151 (52.2)  - | 151 (49.7)  153 (50.3)  - | 0.68 | 287 (38.8)  453 (61.2)  - | 148 (42.8)  197 (56.9)  1 (0.3) | 0.21 |
| Menopausal status  Premenopausal  Postmenopausal | 152 (52.2)  139 (47.8) | 169 (55.6)  135 (44.4) | 0.46 | 332 (44.9)  408 (55.1) | 170 (49.1)  176 (50.9) | 0.19 |
| Type of surgery  MRM  Breast conserving  Missing data | 224 (77.0)  67 (23.0)  - | 227 (74.7)  77 (25.3)  - | 0.57 | 482 (65.1)  258 (34.9)  - | 226 (65.3)  119 (34.4)  1 (0.3) | 0.95 |
| Tumor size (cm)  <2  2.1-5  >5  Missing data | 97 (33.3)  144 (49.5)  50 (17.2)  - | 88 (28.9)  175 (57.6)  41 (13.5)  - | 0.13 | 223 (30.1)  440 (59.5)  77 (10.4)  - | 123 (35.5)  184 (53.2)  36 (10.4)  3 (0.9) | 0.15 |
| Number of positive nodes  0-3  >4 | 67 (23.0)  224 (77.0) | 96 (31.6)  208 (68.4) | **0.022** | 341 (46.1)  399 (53.9) | 185 (53.5)  161 (46.5) | **0.027** |
| Histological grade  I-II  III-Undifferentiated  Missing data | 146 (50.2)  145 (49.8)  - | 144 (47.4)  159 (52.3)  1 (0.3) | 0.57 | 367 (49.6)  373 (50.4)  - | 196 (56.6)  148 (42.8)  2 (0.6) | **0.026** |
| Histology classification  Ductal  Lobular  Mixed  Other  Missing data | 214 (73.5)  35 (12.0)  27 (9.3)  15 (5.2)  - | 222 (73.0)  30 (9.9)  28 (9.2)  16 (5.3)  8 (2.6) | 0.91 | 586 (79.2)  70 (9.5)  46 (6.2)  38 (5.1)  - | 263 (76.0)  38 (11.0)  18 (5.2)  26 (7.5)  1 (0.3) | 0.32 |
| Radiotherapy  Hormonal therapy | 237 (81.4)  260 (89.3) | 224 (73.7)  265 (87.2) | **0.024**  0.45 | 545 (73.6)  539 (72.8) | 231 (66.8)  240 (69.4) | **0.021**  0.25 |

Significant p values are shown in bold.

**Table S2.** Association of CEP17, *HER2* and *TOP2A* markers with basic clinicopathological parameters.

|  | **CEP17** | |  | ***HER2*** | |  | ***TOP2A*** | | |  |
| --- | --- | --- | --- | --- | --- | --- | --- | --- | --- | --- |
|  | **No Gain** | **Gain** | **p** | **Non-amplified** | **Amplified** | **p** | **Deleted** | **Non-amplified** | **Amplified** | **p** |
|  | **N (%)** | **N (%)** |  | **N (%)** | **N (%)** |  | **N (%)** | **N (%)** | **N (%)** |  |
| Randomization group  E-T-CMF  E-CMF  ET-CMF | 306 (49.4)  98 (15.8)  216 (34.8) | 198 (48.2)  59 (14.4)  154 (37.5) | 0.64 | 378 (48.0)  124 (15.8)  285 (36.2) | 126 (51.6)  33 (13.5)  85 (34.8) | 0.55 | 27 (51.9)  11 (21.2)  14 (26.9) | 419 (47.9)  137 (15.7)  319 (36.5) | 58 (55.8)  9 (8.7)  37 (35.6) | 0.15 |
| Menopausal status  Premenopausal  Postmenopausal | 322 (51.9)  298 (48.1) | 162 (39.4)  249 (60.6) | **<0.001** | 367 (46.6)  420 (53.4) | 117 (48.0)  127 (52.0) | 0.77 | 22 (42.3)  30 (57.7) | 416 (47.5)  459 (52.5) | 46 (44.2)  58 (55.8) | 0.64 |
| Tumor size (cm)  <2  2.1-5  >5 | 206 (33.2)  341 (55.0)  73 (11.8) | 114 (27.7)  243 (59.1)  54 (13.1) | 0.17 | 256 (32.5)  435 (55.3)  96 (12.2) | 64 (26.2)  149 (61.1)  31 (12.7) | 0.17 | 12 (23.1)  35 (67.3)  5 (9.6) | 281 (32.1)  485 (55.4)  109 (12.5) | 27 (26.0)  64 (61.5)  13 (12.5) | 0.37 |
| Number of positive nodes  0-3  >4 | 249 (40.2)  371 (59.8) | 159 (38.7)  252 (61.3) | 0.65 | 322 (40.9)  465 (59.1) | 86 (35.2)  158 (64.8) | 0.12 | 14 (26.9)  38 (73.1) | 352 (40.2)  523 (59.8) | 42 (40.4)  62 (59.6) | 0.16 |
| Histological grade  I-II  III-Undifferentiated | 323 (52.1)  297 (47.9) | 190 (46.2)  221 (53.8) | 0.066 | 430 (54.6)  357 (45.4) | 83 (34.0)  161 (66.0) | **<0.001** | 22 (42.3)  30 (57.7) | 451 (51.5)  424 (48.5) | 40 (38.5)  64 (61.5) | **0.023** |
| Histology classification  Ductal  Lobular  Mixed  Other | 467 (75.3)  74 (11.9)  46 (7.4)  33 (5.3) | 333 (81.0)  31 (7.5)  27 (6.6)  20 (4.9) | 0.11 | 588 (74.7)  95 (12.1)  61 (7.8)  43 (5.5) | 212 (86.9)  10 (4.1)  12 (4.9)  10 (4.1) | **<0.001** | 40 (76.9)  7 (13.5)  2 (3.8)  3 (5.8) | 673 (76.9)  90 (10.3)  66 (7.5)  46 (5.3) | 87 (83.7)  8 (7.7)  5 (4.8)  4 (3.8) | 0.69 |
| ER/PgR  Negative  Positive | 143 (23.3)  471 (76.7) | 91 (22.2)  319 (77.8) | 0.71 | 127 (16.2)  655 (83.8) | 107 (44.2)  135 (55.8) | **<0.001** | 19 (37.3)  32 (62.7) | 173 (19.9)  696 (80.1) | 42 (40.4)  62 (59.6) | **<0.001** |
| Ki67  Low  High | 198 (33.2)  398 (66.8) | 124 (30.7)  280 (69.3) | 0.41 | 273 (35.8)  489 (64.2) | 49 (20.6)  189 (79.4) | **<0.001** | 11 (22.0)  39 (78.0) | 282 (33.2)  567 (66.8) | 29 (28.7)  72 (71.3) | 0.19 |

Significant p values are shown in bold.

**Table S3**. Distribution of *HER2* gene status according to HER2 protein expression.

|  | **HER2 (IHC)** | | | |  |
| --- | --- | --- | --- | --- | --- |
|  | 0 | 1+ | 2+ | 3+ | Total |
| ***HER2* (FISH)** | N (%) | N (%) | N (%) | N (%) | N (%) |
| Non-amplified | 312 (97.8) | 359 (94.7) | 98 (57.3) | 3 (2.1) | *772 (76.1)* |
| Amplified | 7 (2.2) | 20 (5.3) | 73 (42.7) | 142 (97.9) | *242 (23.9)* |
| *Total* | *319 (100)* | *379 (100)* | *171 (100)* | *145 (100)* | *1014 (100)* |

**Table S4**. Distribution of *TOP2A* gene status according to TopoIIa protein expression.

|  | **TopoIIa (IHC)** | |  |
| --- | --- | --- | --- |
|  | Negative | Positive | Total |
| ***TOP2A* (FISH)** | N (%) | N (%) | N (%) |
| Deletion | 19 (4.3) | 28 (5.5) | *47 (4.9)* |
| Non-amplified | 384 (87.1) | 425 (83.0) | *809 (84.9)* |
| Amplified | 38 (8.6) | 59 (11.5) | *97 (10.2)* |
| *Total* | *441 (100)* | *512 (100)* | *953 (100)* |

**Table S5**. Distribution of CEP17 status according to HER2 and TopoIIa protein expression.

|  | **HER2 (IHC)** | | | |  | **TopoIIa (IHC)** | |  |
| --- | --- | --- | --- | --- | --- | --- | --- | --- |
|  | 0 | 1+ | 2+ | 3+ | p | Negative | Positive | p |
| **CEP17 (FISH)** | N (%) | N (%) | N (%) | N (%) | <0.001 | N (%) | N (%) | 0.004 |
| No gain | 229 (71.8) | 234 (61.7) | 72 (42.1) | 73 (50.3) |  | 284 (64.4) | 282 (55.1) |  |
| Gain | 90 (28.2) | 145 (38.3) | 99 (57.9) | 72 (49.7) |  | 157 (35.6) | 230 (44.9) |  |
|  |  |  |  |  |  |  |  |  |

**Table S6. Association of *HER2* and *TOP2A* gene status assessed by FISH.**

|  | ***HER2* (FISH)** | |  |
| --- | --- | --- | --- |
|  | Non-amplified | Amplified | Total |
| ***TOP2A* (FISH)** | N (%) | N (%) | N (%) |
| Deletion | 28 (3.6) | 24 (9.8) | *52 (5.0)* |
| Non-amplified | 757 (96.2) | 118 (48.4) | *875 (84.9)* |
| Amplified | 2 (0.3) | 102 (41.8) | *104 (10.1)* |
| *Total* | *787 (100)* | *244 (100)* | *1031 (100)* |

**Table S7.** Survival data for the total study population and according to randomization group.

|  | Total cohort | E-T-CMF | E-CMF | ET-CMF | Log-rank p |
| --- | --- | --- | --- | --- | --- |
|  | N=1031 | N=504 | N=157 | N=370 |  |
| **Disease-free survival (DFS)** |  |  |  |  | 0.32 |
| Events (n, %) | 370 (35.9%) | 176 (34.9%) | 71 (45.2%) | 123 (33.2%) |  |
| Progressions (n, %) | 309 (30.0%) | 147 (29.2%) | 58 (36.9%) | 104 (28.1%) |  |
| 5-year DFS rate (95% CI) | 73.6 (70.9-76.3) | 74.5 (70.6-78.4) | 68.5 (61.2-75.8) | 74.5 (70.0-79.0) |  |
|  |  |  |  |  |  |
| **Overall survival (OS)** |  |  |  |  | 0.17 |
| Deaths (n, %) | 270 (26.2%) | 124 (24.6%) | 57 (36.3%) | 89 (24.1%) |  |
| 5-year OS rate (95% CI) | 86.4 (84.2-88.6) | 88.4 (85.7-91.1) | 80.7 (74.4-87.0) | 86.0 (82.5-89.5) |  |
|  |  |  |  |  |  |
| Median follow-up (months) | 105.6 | 106.6  140.26 | 140.2 | 98.6 |  |
| Range | 0.10-166.7 | 0.10-166.7 | 7.0-162.8 | 0.10-132.5 |  |
|  |  |  |  |  |  |
